# Deleterious effects of polypropylene released from paper cups on blood profile and liver tissue of *Clarias gariepinus:* bioremediation using *Spirulina*

**DOI:** 10.3389/fphys.2024.1380652

**Published:** 2024-05-22

**Authors:** Zainab Eid, Usama M. Mahmoud, Alaa El-Din H. Sayed

**Affiliations:** ^1^ Zoology Department, Faculty of Science, Assiut University, Assiut, Egypt; ^2^ Molecular Biology Research & Studies Institute, Assiut University, Assiut, Egypt

**Keywords:** polypropylene, hemato-biochemical, liver, fish, *Spirulina*

## Abstract

Despite numerous studies on microplastics, the biological impacts of polypropylene microplastics (PP-MPs) and its toxicity on freshwater fish have yet to be fully revealed. The purpose of this research was to look at the potentially harmful effects of PP-MPs in freshwater African catfish *Clarias gariepinus* and bioremediation using *Spirulina*. After acclimatization to laboratory conditions, 108 fish (125 ± 3 gm and 27 ± 2 cm) were assigned into triplicate six experimental groups (12 fish/group), a control group, *Spirulina* group (SP), PP-MP-treated groups (0.14 and 0.28 mg/l PP-MPs), and PP-MP + *Spirulina*-treated groups (0.14 mg/l PP-MPs + 200 mg/L SP and 0.28 mg/l PP-MPs +200 mg/L SP) for 15-day exposure and 45-day recovery after that. The hematological parameters exhibiting significance (RBCs, Hct, Hb, and MCV) or non-significance (MCH and MCHC) either decreased with the increase in PP-MP doses from 0.0 in the control to 0.28 mg/L red blood cells (RBCs), hematocrit (Hct), mean corpuscular hemoglobin (MCH), mean corpuscular hemoglobin concentration (MCHC), hemoglobin (Hb) and platelets or increased with such an increase in doses (mean corpuscular volume (MCV)). The liver enzyme activity, aspartate aminotransferase (AST), alkaline phosphatase (ALP), and alanine aminotransferase (ALT) exhibited non-significant (*p* ≥ 0.05) or significant (*p* < 0.05) increases in (0.14 and 0.28 mg/L) PP-MP-exposed groups, respectively, except ALP. Furthermore, there was a significant (*p* < 0.05) or non-significant (*p* ≥ 0.05) increase in 0.14 and 0.28 mg/l PP-MP +200 mg/L-exposure groups, respectively, compared to the control group and the same exposure group without *Spirulina*. In comparison to the control group, PP-MPs (0.14 and 0.28 mg/L) induced a significant (*p* < 0.05) increase in the percentage of poikilocytosis and nuclear abnormalities of RBCs. The liver tissue from fish exposed to PP-MPs exhibited varying degrees of pathological changes. These results indicated that these pathological changes increased with PP-MP concentration, suggesting that the effect of PP-MPs was dose-dependent. After 45 days of recovery under normal conditions, it was obvious that there was a significant improvement in the percentage of poikilocytosis and nuclear abnormalities of RBCs, as well as a non-significant improvement in hemato-biochemical parameters and liver tissue.

## 1 Introduction

Plastic debris is a significant concern to marine and freshwater ecosystems, acting as a vector for microorganisms (Lobelle and Cunliffe, 2011), a source of gastrointestinal tract (GIT) blockage (Jabeen et al., 2018), and a cause of death (Setälä et al., 2014), eventually affecting humans. Plastic polymers of many different types are produced and released into the environment. In Europe, polyethylene (PE) accounted for 28% of the total output, with PP accounting for 19%, polyvinylchloride (PVC) accounting for 10%, and polystyrene accounting for 7% (Europe, 2017). Considering that PP is the second-most popular plastic resin in terms of manufacturing volume in Europe and worldwide, this is surprising (Statista, 2021), and PP is among the most frequently discovered microplastics in the environment (Peng et al., 2020). PP is a suitable thermoplastic polymer with excellent qualities such as dimensional solidity, fire resistance, simplicity, and a high thermal distortion temperature (Alsabri et al., 2022). PP accounts for 16% of the global plastics industry and is frequently used to manufacture end products such as plastic packaging for customers (Bora et al., 2020). PP has potential applications in the areas of powder technology, composite biotechnology, catalysis, optoelectronics, wastewater treatment (Pankhania et al., 1994), surface coating technology (Huang et al., 2007), flame retardants (Shao et al., 2014), food packaging (Ramos et al., 2012), medical purposes (Martakis et al., 1994), and toys (Specht, 2011). PP is primarily integrated in these goods by molding or extrusion procedures (Zhang and Horrocks, 2003).

According to De Sá et al. (2018), PP was included in just 12.1% of the 157 peer-reviewed ecotoxicity studies published in 2018 involving 612 different microplastics on aquatic species. However, it can be concluded that both PP fibers and fragments at levels observed in the environment have the potential to harm organisms (Horn et al., 2020; Kim et al., 2020). PP was discovered in 1954, and because it has the lowest density among plastics, it was soon adopted (Andrady and Neal, 2009). It is classified as a thermoplastic because, when heated to its melting point, it transforms into a liquid (Papageorgiou et al., 2015). PP is regarded as one of the most promising commodities due to its physical qualities, versatility, and ecologically benign record as a thermoplastic polymer (Ramesh and Vinodh, 2020). PP sales are expected to increase at an annual pace of 6.3% between 2013 and 2019 (Papageorgiou et al., 2015). Furthermore, by 2019, PP revenues are predicted to exceed $124.01 billion, with a 6.3% annual growth rate (Papageorgiou et al., 2015). According to Thummabancha et al. (2016), PP can be classified into three categories depending on its physical and chemical properties: homopolymer (HPP) is semi-crystalline and composed of just propylene monomers; random copolymer (RCP) comprises ethylene as a co-monomer in the PP chains at levels between 1% and 8%; and finally, impact copolymer (ICP) incorporates a 45%–65% ethylene co-mixed RCP phase. In the plastics industry, HPP is the most often used PP. According to one study, the market share for various types of PP is as follows: HPP 65%–75%, ICP 20%–30%, and RCP 5%–10% (Maddah, 2016).

It is known that according to Food and Drug Administration (FDA) guidelines, PP particles are safe polymers (Haya and Maher, 2013); however, other research studies show that heat (Song et al., 2003) and propylene gas (Quest et al., 1984) are the primary reasons for their toxicity. Despite the fact that much research has been undertaken to evaluate propylene gas-related toxicity, there are few studies that estimate the direct toxicity of PP particles (Bobori et al., 2022). Some studies have suggested that the potential toxicity of microplastic particles, such as hemolytic effects on red blood cells (RBCs), can be controlled by concentration, exposure period, shape, and surface charge (Hamed et al., 2019a; Hwang et al., 2019; Sayed et al., 2021a; Hamed et al., 2022; Soliman et al., 2023). As a result, Hwang et al. (2019) investigated RBC hemolysis following direct contact with PP particles of varying sizes (Mazzaglia et al., 2018). PP have some negative effects such as gut retention, reduced development and eating rates, changed metabolic rates and processes, alterations to the molt process, reduced reproduction, immune response induction, oxidative stress and antioxidant responses, altered gut microbiota, and (very rarely) mortality (De Sá et al., 2018; do Nascimento et al., 2023; Jeyavani et al., 2023; Yang et al., 2023; Yedier et al., 2023). As a result, plastic garbage is ubiquitous in the atmosphere, and it has been proposed as an ecological indicator for the “Anthropocene Era” (Zalasiewicz et al., 2016). According to many literature reviews on laboratory ecotoxicity investigations, PP has received least attention (Maity and Pramanick, 2020; Peng et al., 2020).

*Spirulina* is a freshwater single-cell microscopic microalga with antioxidant enzymes that can prevent free radical formation (Seshadri et al., 1991) and an immune stimulant that is cost-effective and has fewer adverse effects when compared to manufactured treatments (Belay, 2020). Furthermore, *Spirulina platensis* (SP) has been demonstrated to protect against the toxicity of various well-known drugs, including aspirin (Mahmoud and Abd El-Ghffar, 2019), methotrexate (Khafaga and El-Sayed, 2018), D-galactosamine, acetaminophen, and rifampicin (Martin and Sabina, 2016). *Spirulina* is high in protein (50%–70% on dry mass basis), carotenoids, chlorophyll, pigments, essential fatty acids (alpha-linolenic, gamma-linolenic, and linoleic acid) (Peiretti and Meineri, 2011), photosynthetic pigments (Bermejo et al., 2008), vitamins (Hoseini et al., 2013), and minerals (Babadzhanov et al., 2004) such as Ca, K, Cr, Cu, Mn, Fe, P, Mg, Na, Zn, and Se, making *Spirulina* an effective feed supplement (Yousefi et al., 2019). *Spirulina* has recently been used successfully in fish farming as part of an integrated strategy for food supplementation and wastewater treatment to control water quality (Zhang et al., 2020; Sayed et al., 2022b). *Spirulina* has been reported to mitigate phytohormone toxicity, such as gibberellic acid, by Sayed et al. (2023a), and its protective role against chronic toxicity of chlorpyrifos in *C. gariepinus* was reported by Mokhbatly et al. (2020). *Spirulina* alleviated alterations caused by the toxicity of hydroxychloroquine in *C. gariepinus* (Sayed et al., 2021b), toxic effects of sodium dodecyl sulfate in the *C. gariepinus* (Sayed and Authman, 2018), and lead nitrate cytotoxicity and genotoxicity in *C. gariepinus* (Hamed et al., 2019b). However, until now, there are no reports about the potential protective role of *Spirulina* against PP-MP toxicity, particularly in economically important fish.

The African catfish (*C. gariepinus*), a widely distributed freshwater fish, can be found in both natural and developed aquatic habitats, according to Mohamed et al. (2022) and Hamed et al. (2023). Additionally, because of its well-documented biology, widespread usage in aquaculture, use as a component of human food, and a good fish model for toxicological, immunological, and histopathological studies (Mekkawy et al., 2010; Osman et al., 2019; Mukherjee et al., 2022), it has been employed as a valuable research tool.

There is a gap in the studies regarding the laboratory ecotoxicity of PP-MPs as microplastics released from paper cups. Therefore, the primary goal of this research is to investigate the effects of PP-MPs on African catfish *C. gariepinus*. More research should be done to determine the environmental risks of PP-MPs released from paper cups as well as how to deal with this new source of environmental burden.

## 2 Materials and methods

### 2.1 Chemicals and *Spirulina platensis*

PP-MPs are microsized, colored, and spherical with a mean size of approximately 35 µm and were purchased from Sigma-Aldrich.

*Spirulina* tablets were purchased from Japan Algae Co., Ltd.

### 2.2 Particle characterization

A methodology for characterizing PP-MPs was carried out via scanning electron microscopy at the scanning electron microscope unit (SEMU), Assiut University. The FTIR spectra were obtained, analyzed, and then compared with a set of reference data to identify the peaks according to Fang et al. (2012) and verify the PP-MPs.

### 2.3 Specimen collection

A juvenile population of African catfish (*C. gariepinus*) was collected from the aquaponic unit and then transported to 100-L tanks filled with dechlorinated water in the Fish Pollution Laboratory at Assiut University and allowed to acclimatize for 4 weeks. Fish were kept under regulated conditions throughout the studies, including 28°C water, 7.4 pH, 18.14 mg L-1 dissolved oxygen, 0.478 μS/cm conductivity, and a 12-h light: dark photoperiod. The fish were fed a commercial pellet diet of 3% of their body weight each day (crude protein, 30%; energy, 3890 kcal/kg; fibers, 5.71%; and crude fat, 3.08) provided by SKRETTING Company, Egypt, and the water was changed every day to get rid of impurities from metabolic waste. The African catfish were examined both before and after acclimatization to guarantee their health according to Batts et al. (2020).

### 2.4 Experimental design

After acclimatization to laboratory conditions, *C. gariepinus* fish (125 ± 3 gm and 27 ± 2 cm) were assigned to six triplicate experimental groups (18 fish/group, 6 fish in each replicate): a control group, a *Spirulina* group (200 mg/L), PP-MP-treated groups (0.14 and 0.28 mg/l PP-MPs), and PP-MP + *Spirulina*-treated groups (0.14 mg/l PP-MPs +200 mg/L SP and 0.28 mg/l PP-MPs +200 mg/L SP) for 15 days. Each group of fish was kept in its own aquarium with 70 L of water, with half of the water changed, and the PP-MP concentrations were reduced every 2 days and 45-day recovery after that. The doses of PP-MPs were selected according to Lei et al. (2018) and Jemec Kokalj et al. (2022) and the *Spirulina* dose according to Sayed and Authman (2018).

### 2.5 Blood sample collection

Six fish were randomly chosen from each group (two fish from each aquarium per sampling period) and subjected to anesthesia using ice at the end of both the exposure and recovery periods to mitigate stress (Wilson et al., 2009). Blood samples were drawn from the caudal veins and placed in heparinized and non-heparinized tubes for hemato-biochemical analysis. The same goes for blood smears, which were created on tidy glass slides and counted in five fields in each of the three slides of each fish group (about 1,500 cells).

### 2.6 Hemato-biochemical parameters

A hemocytometer was used to count the red blood cells (RBCs) (Stevens, 1997). To measure the hematocrit (Hct), Hesser’s microhematocrit method from 1960 was applied (Hesser, 1960). Lee et al. (1999)’s assessment of hemoglobin levels was used. Using the formulas recommended by Lee et al. (1999), the hematimetric indices were calculated from RBC counts, hemoglobin, and hematocrit. Mean corpuscular volume (MCV), mean corpuscular hemoglobin (MCH), and mean corpuscular hemoglobin concentration (MCHC) were calculated using the following equations (Bain et al., 2016):

$$\text{MCV }\left(\mu m^{3}\right)=\frac{\left[\left(\text{Hct},\%\right)\times 10\right]}{\;\left(\text{RBC}\times \frac{\text{Million}}{\;\mu L}\right)},$$

$$\text{MCH }\left(\text{pg}\right)=\frac{\left[\left(\text{Hb},\frac{g}{\text{dL}}\right)\times 10\right]}{\;\left(\text{RBC}\times \frac{\text{Million}}{\;\mu L}\right)},$$

$$\text{MCHC }\left(\%\right)=\frac{\left[\left(\text{Hb},\frac{g}{\text{dL}}\right)\times 100\right]}{\;\left(\text{Hct},\%\right)}.$$

Blood in non-heparinized tubes was allowed to coagulate at 4°C before being separated by centrifugation at 5,000 rpm for 20 min at 4°C to isolate the serum. Using kits from Bio Diagnostic in Egypt, the biochemical parameters such as alkaline phosphatase (ALP), aspartic aminotransferase (AST), and alanine aminotransferase (ALT) were measured.

### 2.7 Histological and histopathological assessment

Following exposure and recovery, the liver of *C. gariepinus* was dissected and preserved in a 10% neutral formalin solution. These samples were then dehydrated using a graded ethanol series (70%, 75%, 80%, 90%, and 100%). The samples were embedded in paraffin at 62°C and cut into 5-µm-thick sections with a precision microtome (MT-990; RMC Products, United States of America). Harris’ hematoxylin and eosin staining was used on the sections (Biancotti et al., 2020). The samples were examined using an Olympus BX-51 light microscope (Olympus Corp., Japan) in conjunction with an ARTCAM-150 PIII digital camera.

### 2.8 Statistical analysis

The fundamental statistics, means, standard errors, and ranges were estimated. The pattern of variation was investigated using one-way analysis of variance with the SPSS software (IBM-SPSS, 2012) at a significance level of 0.05. For multiple comparisons, the Tukey’s HSD test and Dunnett’s test were used.

## 3 Results

### 3.1 Characterization of PP-MP particles

**FIGURE 1:**
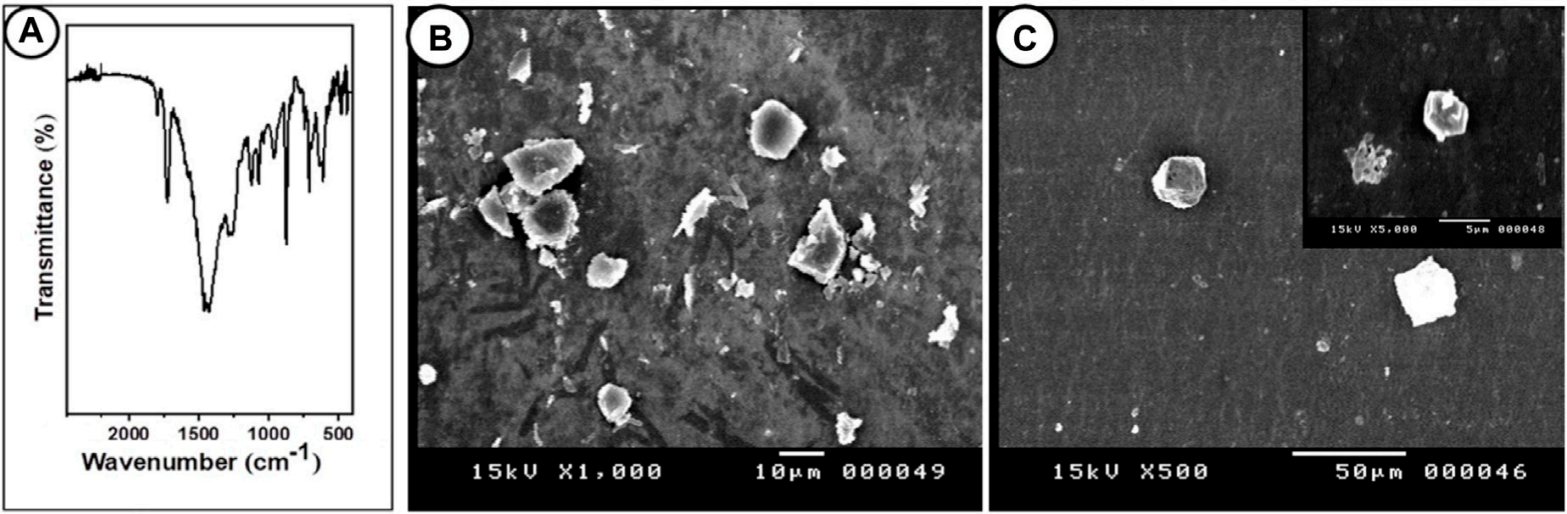
FTIR and SEM images of PP particles. **(A)** Fourier-transform infrared spectroscopy (FTIR) of polypropylene microplastics, and **(B, C)** SEM images of PP particles (scale bar = 10 μm).

The morphology of the PP-MP particles was confirmed by the SEM images; different spherical forms were seen (Figures 1B, C). The detection of chemical bonding in PP-MP plastics was aided by FTIR spectroscopy (Figure 1A). Similar peak values were seen in the FTIR spectra for PP-MPs at 3426 cm^-1^ (O–H stretching vibration), 2,957 cm^-^1 (CH_3_ asymmetrical stretching), 2,918 cm^-1^ (CH_2_ asymmetrical stretching), 2,850 cm^-1^ (CH_2_ stretching), 1,463 cm^-^1 (CH_3_ symmetrical bending), 1,290 cm^-^1 (CH_3_ rocking), 1,150 cm^-^1 (C–H wagging), 964 cm^-1^ (CH_3_ rocking), and 839 cm^-1^ (C–H rocking), and no extra bands related to impurities were detected, which confirms the high purity of this sample. The FTIR peak value of PP-MP polymers was reported by Tiwari et al. (2019) and Sathish et al. (2020), which is consistent with our results.

### 3.2 Effects on hemato-biochemical parameters

The hematological parameters of *C*. *gariepinus* exposed to PP-MPs (0.14 mg/L), PP-MPs (0.28 mg/L), PP-MP (0.14 mg/L) + *Spirulina* (SP) (200 mg/L), PP-MP (0.28 mg/L) + *Spirulina* (200 mg/L), and *Spirulina* (200 mg/L) as a positive control for 15 days are documented in Table 1. Except for MCV, MCH, and MCHC, all parameters showed significant differences (*p* < 0.05) between the treated groups and the control group. The hematological parameters exhibiting significance (RBCs, Hct, Hb, and MCV) or non-significance (MCH and MCHC) either decreased with the increase in PP-MP doses from 0.0 to 0.28 mg/L in the control group (RBCs, Hct, MCH, MCHC, Hb, and platelets) or increased with such an increase in doses (MCV), as shown in Table 1. In the 200 mg/L SP-exposed group, there was a non-significant (*p* ≥ 0.05) increase or decrease in all parameters compared to the control group. Furthermore, in the 0.14 mg/l PP-MP +200 mg/L SP-exposed group, there was a non-significant increase (*p* ≥ 0.05) in all parameters compared to the control and the same exposed group without *Spirulina,* except Hb. Moreover, in the 0.28 mg/l PP-MP +200 mg/L SP-exposed group, there was a significant decrease (*p* < 0.05) compared to the control group and non-significant (*p* ≥ 0.05) increase compared to the same exposed group without *Spirulina* in all parameters, except MCV and MCH. The liver enzyme activities, particularly of aspartate aminotransferase (AST), alkaline phosphatase (ALP), and alanine aminotransferase (ALT), are recorded in Table 1. The parameters (AST, ALT, and ALP) exhibiting a non-significant (*p* ≥ 0.05) increase in the PP-MP (0.14 mg/L)-exposed group and a significant increase in the PP-MP (0.28 mg/L)-exposed group except (ALP) were significantly decreased compared to the control group. In the 200 mg/L SP-exposed group, there was a non-significant (*p* ≥ 0.05) increase or decrease in these parameters compared to the control group. Furthermore, in the 0.14 mg/l PP-MP +200 mg/L SP-exposed group, there was a significant increase (*p* < 0.05) in all parameters compared to the control group and the same exposed group without *Spirulina*. Moreover, in the 0.28 mg/l PP-MP +200 mg/L SP-exposed group, there was a non-significant (*p* ≥ 0.05) increase compared to the control group and non-significant decrease compared to the same exposed group without *Spirulina* in all parameters except ALP, as shown in Table 1.

**TABLE 1:** Hematological and biochemical parameters (liver enzyme activity) of African catfish (*C*. *gariepinus*) exposed to PP-MPs (0.14 mg/L), PP-MPs (0.28 mg/L), PP-MP (0.14 mg/L) + *Spirulina* (200 mg/L), PP-MP (0.28 mg/L) + *Spirulina* (200 mg/L), and *Spirulina* (200 mg/L) (as a positive control) for 15 days.

Treatment parameter	Control	0.14 mg/l PP-MPs	0.28 mg/l PP-MPs	200 mg/L SP	0.14 mg/l PP-MP + 200 mg/L SP	0.28 mg/l PP-MP + 200 mg/L SP
	Mean ± SE (min–max)	Mean ± SE (min–max)	Mean ± SE (min–max)	Mean ± SE (min–max)	Mean ± SE (min–max)	Mean ± SE (min–max)
Hematological parameters						
RBC’s (million/μL)	3.15 ± 0.05^ab^ (3.04–3.4)	2.95 ± 0.04^bc^ (2.87–3.1)	2.55 ± 0.09^d^ (2.35–2.8)	3.29 ± 0.13^a^ (3.07–3.9)	3.25 ± 0.05^a^ (3.16–3.41)	2.74 ± 0.04^cd^ (2.65–2.9)
Hb (Mg/dL)	9.15 ± 0.29^a^ (8.5–10.5)	8.23 ± 0.15^ab^ (7.72–8.5)	7.18 ± 0.17^c^ (6.5–7.8)	9.12 ± 0.29^a^ (8.5–10.5)	9.05 ± 0.16^a^ (8.49–9.35)	7.48 ± 0.40^bc^ (6.57–8.8)
Hct (%)	35.54 ± 0.22^a^ (34.89–36.5)	33.96 ± 0.24^c^ (33.07–34.5)	31.19 ± 0.20^e^ (30.68–32.1)	35.26 ± 0.45^a^ (33.2–36.5)	37.36 ± 0.27^b^ (36.37–37.95)	32.34 ± 0.27^d^ (31.56–33.2)
MCV (µm³)	112.32 ± 1.18^ab^ (107.35–114.84)	114.65 ± 2.37^abc^ (106.66–118.97)	122.1 ± 3.67^bc^ (110.36–130.42)	107.75 ± 4.67^a^ (85.13–114.84)	126.11 ± 2.61^c^ (117.33–130.86)	117.68 ± 1.60^abc^ (110.69–122.59)
MCH (Pg)	28.89 ± 0.61^a^ (26.88–30.88)	27.79 ± 0.87^a^ (24.91–29.31)	28.06 ± 0.82^a^ (25–30)	27.79 ± 1.21^a^ (22.56–30.88)	30.57 ± 0.96^a^ (27.4–32.24)	27.16 ± 1.22^a^ (24.32–31.43)
MCHC (g/dL)	25.62 ± 0.70^ab^ (23.88–28.77)	24.09 ± 0.27^ab^ (23.12–24.64)	22.92 ± 0.65^a^ (20.25–25)	25.81 ± 0.71^ab^ (23.88–28.77)	26.49 ± 0.30^b^ (25.43–27.1)	23.01 ± 1.15^a^ (20.39–26.51)
Platelets (thousands/μL)	220.39 ± 3.19^ab^ (214–235)	210.28 ± 3.85^bc^ (198.99–222)	206.12 ± 1.32^c^ (201–210)	220.96 ± 3.14^ab^ (214–235)	231.3 ± 4.23^a^ (218.89–244.2)	207.62 ± 1.75^c^ (200–212)
Liver enzyme activity						
AST (µ/L)	33.82 ± 0.78^a^ (32.12–36.81)	35.63 ± 0.54^ab^ (33.78–36.91)	36.5 ± 0.38^b^ (35.38–37.8)	33.94 ± 0.75^a^ (32.12–36.81)	39.19 ± 0.60^c^ (37.16–40.6)	35.32 ± 0.55^ab^ (33.71–37.11)
ALT (µ/L)	16.64 ± 0.42^a^ (15.8–18.1)	17.48 ± 0.39^ab^ (16.24–18.5)	18.79 ± 0.25^bc^ (17.8–19.6)	16.97 ± 0.43^a^ (15.8–18.1)	19.23 ± 0.43^c^ (17.86–20.35)	18.06 ± 0.37^abc^ (17.2–19.8)
ALP (µ/L)	46.11 ± 1.29^a^ (43–51)	46.77 ± 0.73^a^ (44.55–49)	40.96 ± 0.93^b^ (38–45)	45.76 ± 1.43^a^ (42–51)	51.44 ± 0.81^c^ (49–53.9)	42.96 ± 1.01^ab^ (41–47)

Values with the same letters within a parameter are not significantly different at the level of 0.05 (horizontal comparison).

After 45 days of recovery, comparable patterns of notable fluctuations toward an increase or decrease were noted in the hematological parameters, except in the 0.28 mg/l PP-MP +200 mg/L SP-exposed group; after the recovery period, there was a non-significant (*p* ≥ 0.05) decrease in all parameters compared to this dose in the exposure period Table 2. The parameters (AST, ALT, and ALP) exhibiting comparable patterns of notable fluctuations toward an increase or decrease except in 0.28 mg/l PP-MP +200 mg/L SP-exposed group showed a significant (*p* < 0.05) increase in these parameters after the recovery period compared to this dose to the same exposed group without *Spirulina* in the exposure period Table 2.

**TABLE 2:** Hematological and biochemical parameters (liver enzyme activity) of African catfish (*C*. *gariepinus*) exposed to PP-MPs (0.14 mg/L), PP-MPs (0.28 mg/L), PP-MP (0.14 mg/L) + *Spirulina* (200 mg/L), PP-MP (0.28 mg/L) + *Spirulina* (200 mg/L), and *Spirulina* (200 mg/L) (as a positive control) after 45 days of recovery.

Treatment parameter	Control	0.14 mg/l PP-MPs	0.28 mg/l PP-MPs	200 mg/L SP	0.14 mg/l PP-MP + 200 mg/L SP	0.28 mg/l PP-MP + 200 mg/L SP
	Mean ± SE (min–max)	Mean ± SE (min–max)	Mean ± SE (min–max)	Mean ± SE (min–max)	Mean ± SE (min–max)	Mean ± SE (min–max)
Hematological parameters						
RBC’s (million/μL)	3.13 ± 0.06^ab^ (3–3.4)	2.92 ± 0.05^bc^ (2.8–3.1)	2.82 ± 0.10^c^ (2.6–3.1)	3.28 ± 0.14^a^ (3–3.9)	3.22 ± 0.05^ab^ (3.1–3.4)	2.72 ± 0.05^c^ (2.6–2.9)
Hb (Mg/dL)	9.05 ± 0.29^a^ (8.4–10.4)	8.12 ± 0.15^ab^ (7.6–8.4)	7.9 ± 0.19^b^ (7.2–8.6)	9.03 ± 0.31^a^ (8.4–10.5)	8.98 ± 0.17^a^ (8.4–9.3)	7.4 ± 0.39^b^ (6.5–8.7)
Hct (%)	35.15 ± 0.22^a^ (34.5–36.1)	33.63 ± 0.25^c^ (32.7–34.2)	34.3 ± 0.22^ac^ (33.7–35.3)	34.95 ± 0.47^a^ (32.9–36.5)	37 ± 0.27^b^ (36–37.6)	32.03 ± 0.28^d^ (31.2–32.9)
MCV (µm³)	111.2 ± 1.17^a^ (106.3–113.7)	113.52 ± 2.34^ab^ (105.6–117.8)	134.32 ± 4.04^c^ (121.4–143.5)	106.87 ± 4.62^a^ (84.3–113.7)	124.88 ± 2.59^bc^ (116.2–129.6)	116.52 ± 1.52^ab^ (109.6–121.4)
MCH (Pg)	28.6 ± 0.61^a^ (26.6–30.6)	27.5 ± 0.86^a^ (24.7–29)	30.87 ± 0.90^a^ (27.5–33)	27.57 ± 1.23^a^ (22.3–30.9)	30.25 ± 0.95^a^ (27.1–31.9)	26.9 ± 1.21^a^ (24.1–31.1)
MCHC (g/dL)	25.37 ± 0.69^ab^ (23.6–28.5)	23.83 ± 0.27^ab^ (22.9–24.4)	25.22 ± 0.71^ab^ (22.3–27.5)	25.6 ± 0.75a^b^ (23.6–28.8)	26.23 ± 0.29^b^ (25.2–26.8)	22.77 ± 1.14^a^ (20.2–26.2)
Platelets (thousands/μL)	218.22 ± 3.16^ab^ (211.9–232.7)	208.18 ± 3.81^bc^ (197–219.8)	226.73 ± 1.45^a^ (221.1–231)	219.17 ± 3.46^ab^ (211.9–235)	229 ± 4.19^a^ (216.7–241.8)	205.55 ± 1.73^c^ (198–209.9)
Liver enzyme activity						
AST (µ/L)	33.47 ± 0.77^a^ (31.8–36.4)	35.27 ± 0.54^a^ (33.4–36.5)	40.15 ± 0.42^b^ (38.9–41.6)	33.63 ± 0.71^a^ (32.1–36.4)	38.8 ± 0.59^b^ (36.8–40.2)	34.97 ± 0.54^a^ (33.4–36.7)
ALT (µ/L)	16.45 ± 0.42^a^ (15.6–17.9)	17.28 ± 0.38^a^ (16.1–18.3)	20.68 ± 0.28^c^ (19.6–21.6)	16.82 ± 0.44^a^ (15.6–17.9)	19.03 ± 0.41^b^ (17.7–20.1)	17.87 ± 0.37^ab^ (17–19.6)
ALP (µ/L)	45.67 ± 1.27^a^ (42.6–50.5)	46.3 ± 0.72^a^ (44.1–48.5)	45.05 ± 1.02^a^ (41.8–49.5)	45.38 ± 1.39^a^ (41.6–50.5)	50.93 ± 0.80^b^ (48.5–53.4)	42.55 ± 0.99^a^ (40.6–46.5)

Values with the same letters within a parameter are not significantly different at the level of 0.05 (horizontal comparison).

### 3.3 Effects on the erythron profiles

**FIGURE 2:**
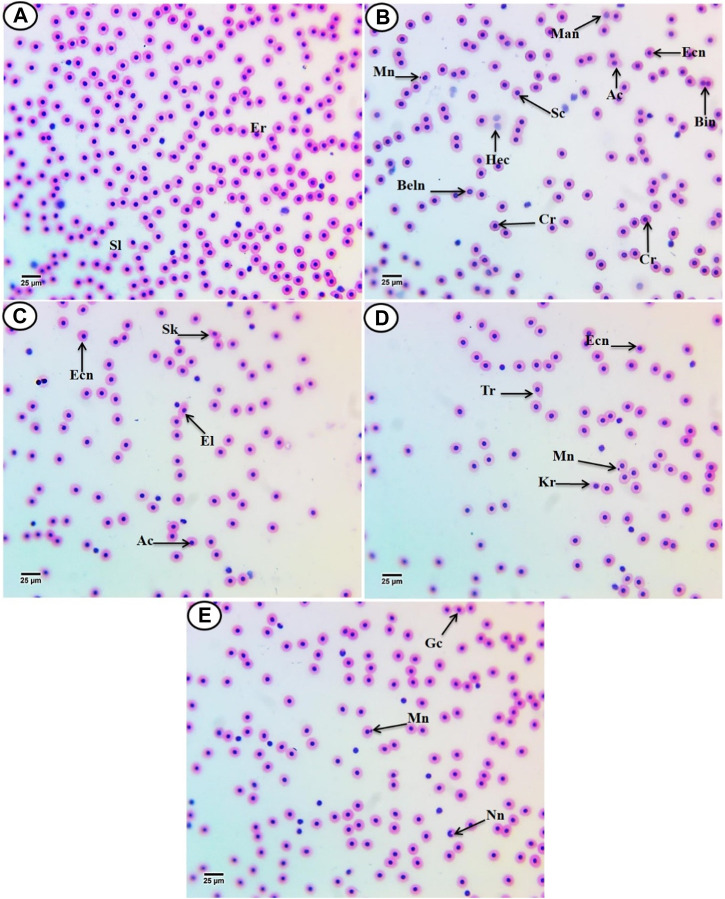
Hematoxylin and eosin-stained blood film from *C*. *gariepinus.* **(A)** Control group showing normal erythrocytes with a rounded-shape nucleus **(Er)** and differential leukocytes (small lymphocytes **(SL)**). [From **(B–E)**] Blood film of treated *C. gariepinus* showing the deformed ones: **Cr**, echinocytes or crenated cell; **Ac**, acanthocytes; **Sk**, sickle cells; **Sc**, swelled cells; **El**, elliptocytes; **Kr**, keratocytes; **Tr**, teardrop-like cells; **Hec**, hemolyzed cells; **Gc**, genuine cell; **Ecn**, eccentric nucleus; **Mac**, macronucleated cells; **Mn**, micronucleus; **Bin**, bionuclei; **Beln**, blebbed nucleus, and **Nn**, notched nucleus. Scale bar = 25 μm.

According to Figure 2A, the erythrocytes (Er) in this species’ blood were spherical and nucleated, with a rounded nucleus positioned in the center. Additionally, based on their size, there were small lymphocytes (SL), with a darkly stained nucleus and a non-granular, weakly basophilic cytoplasm. In the present study, erythrocyte morphological abnormalities were recorded in that species exposed to PP-MPs (0.14 mg/L), PP-MPs (0.28 mg/L), PP-MP (0.14 mg/L) + *Spirulina* (200 mg/L), PP-MP (0.28 mg/L) + *Spirulina* (200 mg/L), and *Spirulina* (200 mg/L) as a positive control for 15 days. These deformations (Figures 2B–E) included echinocytes or crenated cell (Cr), acanthocytes (Ac), sickle cell (Sk), swelled cells (Sc), elliptocytes (EL), keratocytes (Kr), teardrop-like cells (Tr), hemolyzed cells (Hec), genuine cells (Gc), eccentric nucleus (Ecn), macronucleated cells (Mac), micronucleus (Mn), bionuclei (Bin), blebbed nucleus (Beln), and notched nucleus (Nn). PP-MPs (0.14 mg/L and 0.28 mg/L) caused a significant (*p* < 0.05) increase in the percentage of poikilocytosis and nuclear abnormalities of RBCs in relation to the control group, as shown in Table 3. Likewise, there were still significant (*p* < 0.05) increases in the percentage of poikilocytosis and nuclear abnormalities of RBCs in the fish exposed to PP-MPs (0.14 mg/L and 0.28 mg/L) and co-treated with *Spirulina* (200 mg/L) compared to the control group except (Tr, Gc, and Kr) in the PP-MP (0.14 mg/L) + SP (200 mg/L) group, which indicated that the *Spirulina* (200 mg/L) dose was insufficient to treat the effects of PP-MPs. In contrast to fish exposed to PP-MPs alone, fish treated with *Spirulina* showed a significant (*p* < 0.05) decrease in the percentage of poikilocytosis and nuclear abnormalities of RBCs. Significant differences were still evident between the groups after the recovery period in terms of the percentage of poikilocytosis and nuclear abnormalities of RBCs except for Tr in PP-MPs (0.14 mg/L) and PP-MP (0.14 mg/L) + SP (200 mg/L) groups, Kr in PP-MP (0.14 mg/L) + SP (200 mg/L) and PP-MP (0.28 mg/L) + SP (200 mg/L) groups, and the Gc in PP-MP (0.14 mg/L) + SP (200 mg/L) group, demonstrating that the effects of PP-MPs did not entirely disappear after the recovery period, as shown in Table 4. With respect to the control group, the SP (200 mg/L) group showed insignificant variations in most parameters. After 45 days of recovery under normal conditions, the percentage of poikilocytosis and nuclear abnormalities of RBCs showed a noticeable improvement, and the recovery rate was 83.7% with PP-MPs (0.14 mg/L), 83.3% with PP-MPs (0.28 mg/L), 81.4% with PP-MP (0.14 mg/L) + SP (200 mg/L), and 80.6% with PP-MPs] (0.28 mg/L) + SP (200 mg/L) (Table 5), suggesting that the effects of *Spirulina* disappeared after exposure. These differences in the percentage of poikilocytosis and nuclear abnormalities of RBCs increased with PP-MP concentrations, suggesting that the effects of PP-MPs on erythrocyte deformations and nuclear abnormalities were dose-dependent (Tables 3, 4).

**TABLE 3:** Erythrocyte morphological alterations and nuclear abnormalities (mean ± SE and min–max range) in African catfish (*C*. *gariepinus*) after PP-MP exposure for 15 days and treatment with SP (200 mg/L). Significant differences from the control group values were accepted at *p* < 0.05.

Treatment cell type	Control	0.14 mg/l PP-MPs	0.28 mg/l PP-MPs	200 mg/L SP	0.14 mg/l PP-MP + 200 mg/L SP	0.28 mg/l PP-MP + 200 mg/L SP
	Mean ± SE (min–max)	Mean ± SE (min–max)	Mean ± SE (min–max)	Mean ± SE (min–max)	Mean ± SE (min–max)	Mean ± SE (min–max)
Erythrocyte morphological alterations						
Cr	0.36 ± 0.16^a^ (0–3)	4.61 ± 0.27^b^ (2–7)	5.19 ± 0.22^c^ (3–8)	0.73 ± 0.22^a^ (0–5)	4 ± 0.21^b^ (2–7)	4.03 ± 0.23^b^ (1–6)
Ac	0.12 ± 0.09^a^ (0–2)	4.13 ± 0.22^dc^ (2–6)	4.45 ± 0.17^e^ (3-6)	0.48 ± 0.16^b^ (0–4)	3.39 ± 0.19^c^ (1–5)	3.55 ± 0.19^cd^ (1–5)
Tr	0.35 ± 0.18^ab^ (0–3)	1.74 ± 0.23^cd^ (0–4)	2.65 ± 0.19^e^ (1-5)	0.28 ± 0.15^a^ (0–2)	1.26 ± 0.19^bc^ (0–3)	2.03 ± 0.22^de^ (0–4)
Sk	0.87 ± 0.15^a^ (0–2)	3.39 ± 0.19^d^ (2–6)	3.81 ± 0.18^d^ (2–6)	0.18 ± 0.13^b^ (0–2)	2.61 ± 0.16^c^ (1–4)	2.68 ± 0.21^c^ (0–5)
Sc	0.79 ± 0.20^a^ (0–3)	5.45 ± 0.32^c^ (2–9)	5.55 ± 0.32^c^ (0–9)	0.85 ± 0.181^a^ (0–4)	3.55 ± 0.33^b^ (0–8)	3.84 ± 0.34^b^ (2–8)
El	0.16 ± 0.10^a^ (0–2)	2.68 ± 0.24^c^ (1–5)	2.16 ± 0.29^bc^ (0–5)	0.27 ± 0.18^a^ (0–3)	1.97 ± 0.22^bc^ (0–4)	1.45 ± 0.23^b^ (0–4)
Gc	0.47 ± 0.21^ab^ (0–3)	1.81 ± 0.23^bc^ (0–4)	2.29 ± 0.17^c^ (1–4)	0.29 ± 0.12^a^ (0–2)	1.19 ± 0.20^ab^ (0–4)	1.84 ± 0.18^bc^ (0–4)
Kr	0.36 ± 0.16^a^ (0–3)	1.58 ± 0.24^cd^ (0–4)	2.16 ± 0.21^d^ (0–4)	0.33 ± 0.14^a^ (0–2)	1.13 ± 0.19^abc^ (0–3)	1.39 ± 0.17^bc^ (0–3)
Hec	0.49 ± 0.17^a^ (0–3)	5.09 ± 0.25^c^ (3–8)	5.32 ± 0.25^c^ (3–9)	0.77 ± 0.20^a^ (0–4)	3.97 ± 0.25^b^ (1–8)	3.19 ± 0.29^b^ (0–5)
Nuclear abnormalities						
Mn	0.1 ± 0.07^a^ (0–1)	1.9 ± 0.22^c^ (0–4)	2.35 ± 0.22^c^ (0–5)	0.15 ± 0.08^a^ (0–1)	1.26 ± 0.19^b^ (0–3)	1.77 ± 0.16^bc^ (0–3)
Ecn	0.09 ± 0.08^a^ (0–2)	4.32 ± 0.21^d^ (3–7)	4.58 ± 0.20^d^ (3–7)	0.52 ± 0.17^b^ (0–3)	3.35 ± 0.15^c^ (2–5)	3.32 ± 0.26^c^ (1–7)
Nn	0.3 ± 0.16^a^ (0–3)	2.45 ± 0.21^cd^ (0–4)	2.90 ± 0.21^d^ (0–5)	0.12 ± 0.08^a^ (0–1)	1.65 ± 0.21^b^ (0–3)	1.94 ± 0.22^bc^ (0–4)
Man	0.32 ± 0.15^a^ (0–2)	3.39 ± 0.25^c^ (0–5)	3.52 ± 0.23^c^ (0–5)	0.45 ± 0.16^a^ (0–2)	2.06 ± 0.25^b^ (0–4)	2.23 ± 0.26^b^ (0–5)
Bin	0.23 ± 0.11^a^ (0–2)	3.03 ± 0.26^bc^ (0–6)	3.29 ± 0.23^c^ (0–6)	0.21 ± 0.12^a^ (0–3)	2.32 ± 0.24^b^ (0–5)	2.42 ± 0.22^b^ (0–4)
Beln	0.22 ± 0.10^a^ (0–2)	2.26 ± 0.21^bc^ (0–5)	2.68 ± 0.19^c^ (1–5)	0.62 ± 0.15^a^ (0–3)	1.68 ± 0.17^b^ (0–3)	1.74 ± 0.15^b^ (0–3)

Values with the same letters within a parameter are not significantly different at 0.05 level (horizontal comparison).

Cr, echinocytes or crenated cell; Ac, acanthocyte; Sk, sickle cell; Sc, swelled cells; El, elliptocyte; Kr, keratocyte; Tr, teardrop-like cells; Hec, hemolyzed cells; Gc, genuine cell; Ecn, eccentric nucleus; Mac, macronucleated cell; Mn, micronucleus; Bin, bionuclei; Beln, blebbed nucleus; and Nn, notched nucleus.

**TABLE 4:** Erythrocyte morphological alterations and nuclear abnormalities (mean ± SE and min–max range) in African catfish (*C*. *gariepinus*) after PP-MP exposure for 15 days and treatment with SP (200 mg/L) followed by 45 days of recovery. Significant differences from the control group values were accepted at *p* < 0.05.

Treatments cell type	Control	0.14 mg/l PP-MPs	0.28 mg/l PP-MPs	200 mg/L SP	0.14 mg/l PP-MPs + 200 mg/L SP	0.28 mg/l PP-MPs + 200 mg/L SP
	Mean ± SE (min–max)	Mean ± SE (min–max)	Mean ± SE (min–max)	Mean ± SE (min–max)	Mean ± SE (min–max)	Mean ± SE (min–max)
Erythrocyte morphological alterations						
Cr	0.41 ± 0.16^a^ (0–3)	3.84 ± 0.2^b^ (2–6)	4.52 ± 0.15^c^ (3–6)	0.58 ± 0.17^a^ (0–3)	3.29 ± 0.17^b^ (1–5)	3.42 ± 0.17^b^ (1–5)
Ac	0.32 ± 0.16^a^ (0–3)	3.58 ± 0.19^cd^ (2–6)	4.096 ± 0.13^d^ (3–6)	0.44 ± 0.12^a^ (0–2)	2.97 ± .18^b^ (1–5)	3.23 ± 0.16^bc^ (1–5)
Tr	0.42 ± 0.19^ab^ (0–3)	1.48 ± 0.19^bc^ (0–4)	2.35 ± 0.17^d^ (1–4)	0.62 ± 0.15^a^ (0–2)	1.06 ± 0.15^abc^ (0–3)	1.71 ± 0.20^c^ (0–4)
Sk	0.6 ± 0.18^a^ (0–3)	2.58 ± 0.16^c^ (1–4)	3.35 ± 0.13^d^ (2–5)	0.13 ± 0.10^b^ (0–2)	2.06 ± 0.15^c^ (0–3)	2.13 ± 0.21^c^ (0–4)
Sc	0.48 ± 0.18^a^ (0–3)	4.68 ± 0.25^c^ (2–8)	4.9 ± 0.24^c^ (0–7)	0.65 ± 0.14^a^ (0–3)	2.9 ± 0.23^b^ (0–5)	1 ± 0.25 (0–6)^b^
El	0.33 ± 0.09^a^ (0–2)	2.29 ± 0.19^c^ (1–4)	1.74 ± 0.23^c^ (0–4)	0.24 ± 0.15^ab^ (0–2)	1.71 ± 0.21^c^ (0–4)	1.03 ± 0.14^b^ (0–3)
Gc	0.31 ± 0.18^a^ (0–3)	1.35 ± 0.19^bc^ (0–3)	1.87 ± 0.18^c^ (0–4)	0.28 ± 0.12^a^ (0–2)	0.87 ± 0.16^ab^ (0–3)	1.61 ± 0.16^c^ (0–4)
Kr	0.30 ± 0.13^ab^ (0–2)	1.23 ± 0.19^ab^ (0–4)	1.52 ± 0.17^b^ (0–3)	0.34 ± 0.13^a^ (0–2)	0.9 ± 0.15^a^ (0–2)	1.03 ± 0.15^ab^ (0–3)
Hec	0.61 ± 0.21^a^ (0–3)	4.52 ± 0.17^d^ (2–6)	4.42 ± 0.15^d^ (3–6)	0.71 ± 0.18^a^ (0–3)	3.23 ± 0.23^c^ (1–8)	2.35 ± 0.25^b^ (0–5)
Nuclear abnormalities						
Mn	0.08 ± 0.07^a^ (0–1)	1.55 ± 0.196^c^ (0–4)	1.81 ± 0.16^c^ (0–3)	0.13 ± 0.08^a^ (0–1)	1 ± 0.17^b^ (0–3)	1.48 ± 0.12^c^ (0–2)
Ecn	0.081 ± 0.09^a^ (0–2)	3.8 ± 0.16^d^ (2–5)	3.48 ± 0.21^d^ (0–5)	0.55 ± 0.17^a^ (0–3)	2.68 ± 0.16^c^ (1–4)	2.67 ± 0.22^c^ (0–5)
Nn	0.31 ± 0.17^a^ (0–3)	1.93 ± 0.24^bc^ (0–4)	2.38 ± 0.18^c^ (0–4)	0.09 ± 0.07^a^ (0–1)	1.26 ± 0.20^b^ (0–3)	1.55 ± 0.20^b^ (0–3)
Man	0.36 ± 0.17^a^ (0–3)	2.84 ± 0.21^c^ (0–4)	2.87 ± 0.24^c^ (0–5)	0.48 ± 0.16^a^ (0–2)	1.67 ± 0.20^b^ (0–4)	1.77 ± 0.20^b^ (0–3)
Bin	0.24 ± 0.12^a^ (0–2)	2.55 ± 0.18^c^ (0–4)	2.58 ± 0.22^c^ (0–5)	0.19 ± 0.10^a^ (0–2)	1.93 ± 0.24^bc^ (0–5)	1.77 ± 0.20^b^ (0–3)
Beln	0.29 ± 0.16^a^ (0–3)	1.8 ± 0.16^bc^ (0–3)	2.19 ± 0.18^c^ (0–4)	0.29 ± 0.14^a^ (0–2)	1.26 ± 0.15^b^ (0–3)	1.39 ± 0.17 ^b^ (0–3)

Values with the same letters within a parameter are not significantly different at the level of 0.05 (horizontal comparison).

Cr, echinocytes or crenated cell; Ac, acanthocytes; Sk, sickle cells; Sc, swelled cells; El, elliptocytes; Kr, keratocytes; Tr, teardrop-like cells; Hec, hemolyzed cells; Gc, genuine cell; Ecn, eccentric nucleus; Mac, macronucleated cells; Mn, micronucleus; Bin, bionuclei; Beln, blebbed nucleus; and Nn, notched nucleus.

**TABLE 5:** Response of the animals after 45 days of recovery.

Treatment	Total number of exposure alteration	Total number of recovery alteration	Animal response for recovery (%)
Control	150	132↓	88
0.14 mg/l PP-MPs	1,483	1,241 ↓	83.7
0.28 mg/l PP-MPs	1,640	1,367 ↓	83.3
200 mg/L SP	169	156 ↓	92.3
0.14 mg/l PP-MP + 200 mg/L SP	1,097	893 ↓	81.4
0.28 mg/l PP-MP + 200 mg/L SP	1,160	935 ↓	80.6

### 3.4 Effects on the liver tissue and the role of *Spirulina*

**FIGURE 3:**
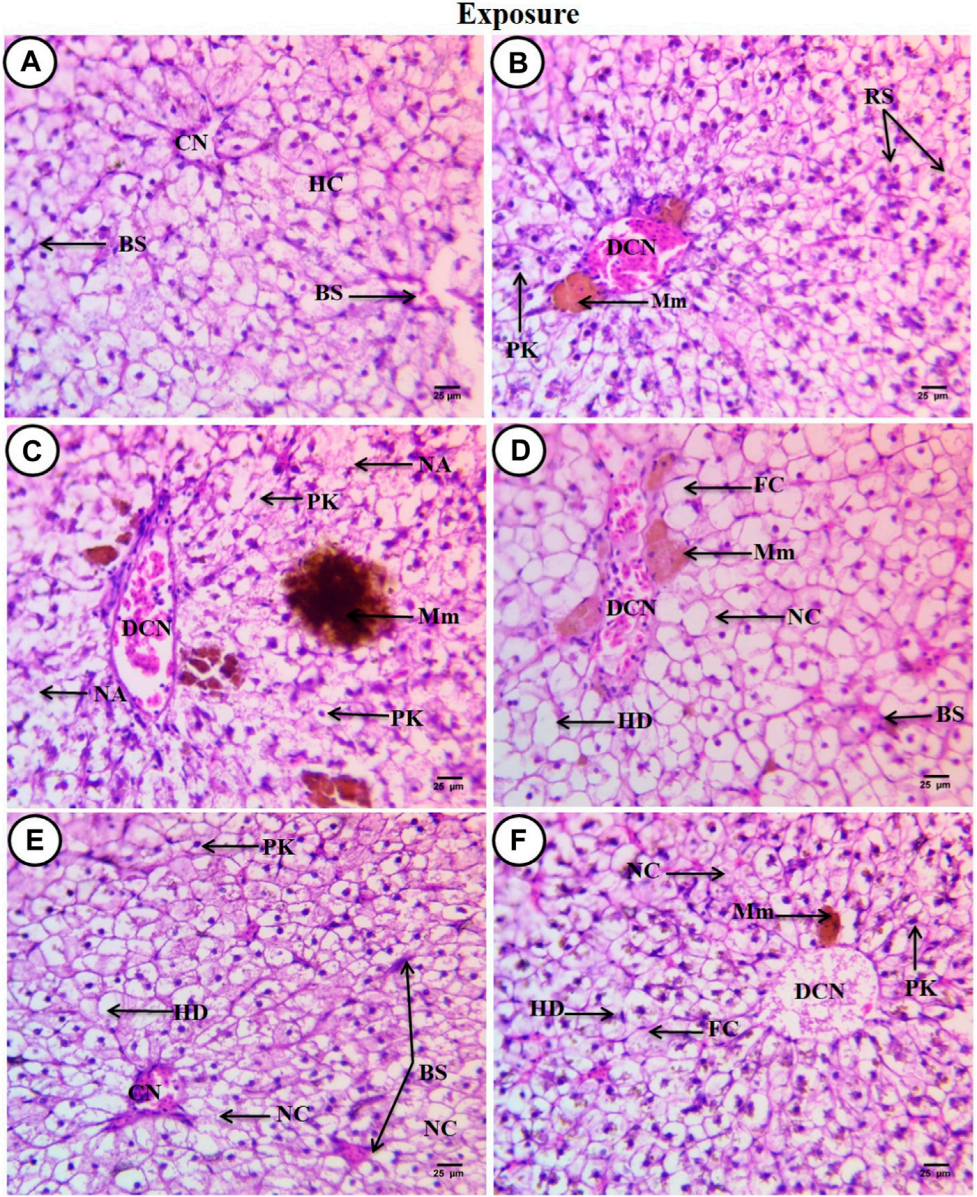
Sections of the liver from control and treated fish stained with hematoxylin and eosin (×400) after an exposure period of 15 days. **(A)** Section from the control fish group revealing typical histological features of the liver tissue of *C. gariepinus*. **(B)** Liver section from the *Spirulina* (200 mg/L)-exposed fish. **(C)** Liver section from a fish exposed to PP (0.28 mg/L) showing varying degrees of damage. **(D)** Liver section from a fish exposed to PP (0.28 mg/L) plus *Spirulina*. **(E)** Liver section from a fish exposed to polypropylene (0.14 mg/L). **(F)** Liver section from a fish exposed to PP (0.14 mg/L) plus *Spirulina*. Labeled structures are as follows: BS, blood sinusoids; CV, central vein; HC, hepatocytes; DCV, dilatation of the central vein; Mm, melanomacrophage centers; PK, pyknotic nucleus; RS, rosette shape hepatocytes; NA, necrotic area; HD, hydropic degeneration; FC, fatty cells with an eccentric flat nucleus, and NC, necrotic cells.

Sections of *C. gariepinus* liver stained with hematoxylin and eosin and treated to SP (200 mg/L), PP-MPs (0.28 mg/L), PP-MP (0.28 mg/L) + SP (200 mg/L), PP-MPs (0.14 mg/L), and PP-MP (0.14 mg/L) + SP (200 mg/L) as a positive control for 15 days are shown in (Figures 3A–E). The control group displayed characteristic *C. gariepinus* liver tissue histological features, such as polygonal and regularly organized wedge-shaped hepatocytes with central nuclei surrounding the central vein and blood sinusoids (Figure 3A). While liver sections from fish treated with SP (200 mg/L) showed a considerably improved tissue structure, some hepatic structure changes were observed, such as a dilated central vein that was full of blood corpuscles, and besides that, there were melanomacrophage centers, hepatocytes aggregated and arranged in rosette shapes with their nuclei in an apical position, and some hepatocytes with pyknotic nuclei (Figure 3B).

In contrast, sections from *C. gariepinus* exposed to PP-MPs (0.28 mg/L) showed varying degrees of damage, including a dilated central vein full of red blood corpuscles and leukocytes, deformation and severe degeneration in hepatic structures, necrosis of main bulk of hepatic cells, vacuolated cells with pyknotic nuclei with fine heterogamous acidophilic in cytoplasm, and increased necrotic area. In addition, it showed the ill-defined hepatocyte boundary or blood sinusoids and increase in the melanomacrophage center in the number and density of the staining degree (Figure 3C). While the PP-MPs (0.28 mg/L) plus *Spirulina*-exposed group showed great amelioration in hepatic structures when compared to the PP-MP (0.28 mg/L)-exposed group without *Spirulina,* some pathological signs were observed such as hydropic degeneration of the main bulk of hepatocytes with a central or eccentric nuclei, few fatty cells with an eccentric flat nucleus, few necrotic areas, a dilated central vein with different types of blood corpuscles, and beside it lie patches of melanomacrophage centers with yellow pigments (Figure 3D). The liver tissue from the PP-MP (0.14 mg/L)-exposed group exhibited little pathological changes compared to the PP-MP (0.28 mg/L)-exposed group, such as few aggregated cells completely lost their cytoplasmic materials and nuclei or necrosed, enlargement of cells around the central vein which was full of blood corpuscles, other cells appeared polygonal or hydropic degenerated with central, eccentric, or pyknotic nuclei, and shrunken blood sinusoids were noticed (Figure 3E). While liver sections from fish exposed to PP-MPs (0.14 mg/L) plus *Spirulina* also showed little amelioration in hepatic structure changes when compared with the same exposed group without *Spirulina*, there were pathological signs like dilated central veins which contained degenerated blood corpuscles (debris), surrounded by small-sized hepatocytes, and, beside them, small melanomacrophage centers. In addition, there was an increase in dispersed pigment granules inside hepatocytes, hydropic degeneration with or without nuclei, while other cells completely necrosed with an ill-defined boundary or contained pyknotic nuclei. A few necrotic areas and fatty cells were observed (Figure 3F).

**FIGURE 4:**
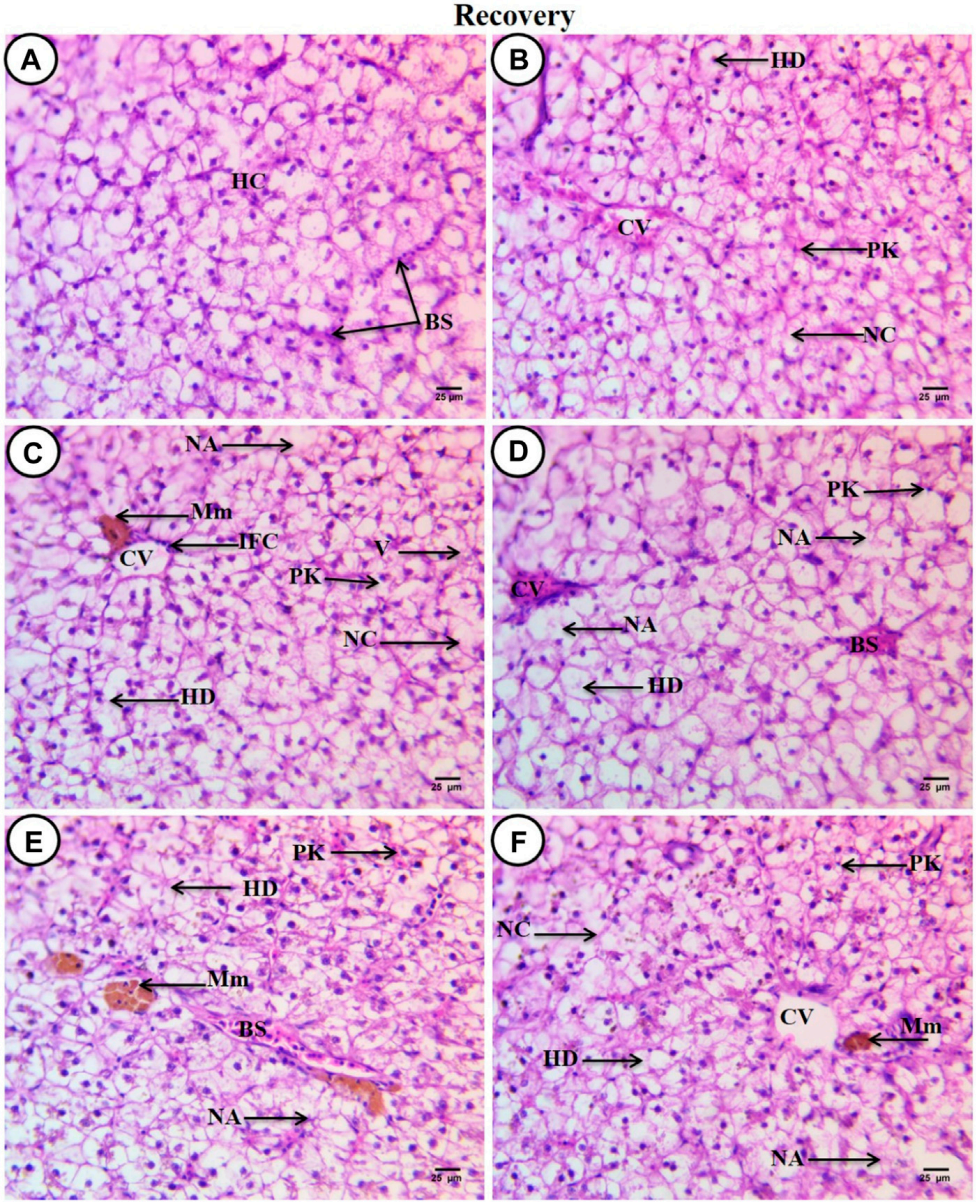
Sections of liver from control and treated fish stained with hematoxylin and eosin (×400) after a recovery period of 45 days. **(A)** Section from the control fish group revealing typical histological features of the liver tissue of *C. gariepinus*. **(B)** Liver section from a fish exposed to *Spirulina* (200 mg/L) after a recovery period of 45 days. **(C)** Liver section from a fish exposed to PP (0.28 mg/L) after a recovery period of 45 days showing great amelioration in hepatic structures. **(D)** Liver section from a fish exposed to PP (0.28 mg/L) plus *Spirulina* after a recovery period of 45 days. **(E)** Liver section from a fish exposed to PP (0.14 mg/L) after a recovery period for 45 days showing little improvement. **(F)** Liver section from a fish exposed to PP (0.14 mg/L) plus *Spirulina* after a recovery period of 45 days showing more or less amelioration. Labeled structures are as follows: BS, blood sinusoids; HC, hepatocytes; CV, central vein; PK, pyknotic nuclei; NC, necrotic cells; HD, hydropic degeneration; NA, necrotic area; IFC, inflammatory cells; V, vacuolated cell, and Mm, melanomacrophage centers.

After a recovery period of 45 days, the control group displayed characteristic C. gariepinus liver tissue histological features (Figure 4A). Liver sections from fish exposed to SP (200 mg/L SP) showed more or less improvement when compared with the same exposed group, although some hepatic structures changed, such as a dilated central vein full of blood corpuscles, aggregated hepatocytes, hydropic degeneration with pyknotic nuclei, the presence of necrotic areas, and increased small-sized blood sinusoids (Figure 4B). Liver sections from fish exposed to PP-MPs (0.28 mg/L) after the recovery period showed great amelioration in hepatic structures when compared with the same exposed group, such as disappearance of blood vessel dilation, decrease in melanomacrophage centers and necrotic areas, and central veins surrounded by few inflammatory cells. Although some cellular hydropic degeneration was observed in many hepatocytes with eccentric nuclei located beside the blood sinusoids, only a few vacuolated and necrotic cells and other cells contained pyknotic nuclei (Figure 4C). While the PP-MP (0.28 mg/L) plus *Spirulina*-exposed group after the recovery period showed great amelioration in hepatic structures when compared with the same group without *Spirulina*, less improvement was noticed when compared with the same exposed group. Still, there were some pathological signs, such as congested blood sinusoids, hydropic degeneration of hepatocytes with clear contours and central or eccentric nuclei, and other cells containing pyknotic nuclei (Figure 4D). The liver tissue from the PP-MP (0.14 mg/L)-exposed group after the recovery period exhibited little improvement, and many cells contained mitotic nuclei when compared with the same exposed group, but some pathological changes were still present, such as an increase in the size of congested blood sinusoids and melanomacrophage centers, many necrotic hepatocytes, hydropic degeneration, and few cells containing vesicular or pyknotic nuclei (Figure 4E). Moreover, the PP-MPs (0.14 mg/L) plus *Spirulina*-exposed group after the recovery period exhibited amelioration when compared with the same exposed group. In addition, some marked hepatic structures changes were observed, such as hydropic degeneration with pyknotic nuclei, other cells completely necrosed with ill-defined boundaries, few necrotic areas, small melanomacrophage centers and dispersed pigment granules inside hepatocytes, and increase in the number of small blood sinusoids (Figure 4F).

## 4 Discussion

Environmental contaminants in the aquatic surroundings disrupt osmotic pressure and fish metabolism as well as have an impact on hematological parameters such as RBC counts, Hb, and Ht levels (Lee et al., 2023). As a result, vital information regarding the general health of fish that have been subjected to environmental stress can be obtained by studying their blood (Burgos-Aceves et al., 2019; Kim et al., 2021a). For instance, blood flow and composition may be considerably affected once MPs are translocated to the circulatory system (Scanes et al., 2019). According to Hirt and Body-Malapel (2020), MPs interact with the fish intestine and can reach the bloodstream, causing systemic problems. As stated by Thummabancha et al. (2016), hematological indices are the primary indicators of fish health. In this study, the parameters (RBCs, Hct, and Hb) exhibited a significance decrease with the increase in PP-MP doses from 0.0 to 0.28 mg/L in the control group. These findings were corroborated with those of Kim et al. (2021b), Hamed et al. (2019a), and Hamed et al. (2022). In the presence of MPs, similar results were obtained with Nile tilapia (*Oreochromis niloticus*) (Hamed et al., 2019a). According to Choi et al. (2021), MP concentrations in the blood lead to higher levels of hemolysis because the sharp surfaces of MPs physically disrupt RBC membranes and result in cell lysis. Additionally, *O. niloticus’s* RBC count, Hb, and Ht levels significantly decreased after exposure to MPs, according to studies by Hamed et al. (2019a) and Ismail et al. (2021). These studies hypothesized that the changes in hematological parameters were caused by MP toxicity, which also caused damage to hematopoietic tissue and RBC hemolysis. Accordingly, the observed alterations in RBC count, Hb, and Ht levels in the current study were probably caused by blood hemolysis and blood cell death brought on by PP-MPs toxicity, which led to anemia, as explained by Lee et al. (2023), after the exposure of *Pseudobagrus fulvidraco* to PE-MPs. The results in the MCV, MCH, and MCHC did not show any statistically significant changes (*p* < 0.05) between control fish and those treated with the two dosages of microplastics of 0.28 and 0.14 mg/L of PP-MPs (Table 1). Nascimento et al. (2023) found comparable results, observing non-significant statistical differences between control Nile tilapia (*O. niloticus*) and those treated with two doses of microplastics of 100 and 500 μg of PP-MPs. Erythrocyte degeneration has been identified as a pathological state in fish exposed to toxicants (Adeyemo, 2005). Erythrocytes have a critical role in oxygen transport (Benedik and Hamlin, 2014). Erythrocyte deformations result in low oxygen levels, which disrupt the circulatory system and induce respiratory dysfunction, both of which can modify erythrocyte morphology (Brown et al., 1994; Benedik and Hamlin, 2014). In the present work, the blood cell alterations of varying degrees were observed in *C. gariepinus*, depending on the concentration of PP-MPs and co-treatment with *Spirulina*. Similar findings were mentioned by Hamed et al. (2022), Hamed et al. (2021), and Sayed et al. (2021a) induced by polyethylene polymers of various sizes (nano, micro, and macro) in juveniles, *Cyprinus carpio*. Numerous explanations have been offered to explain the morphological alterations in blood cells induced by stress (Harper and Wolf, 2009; Mekkawy et al., 2011). There are various ways that red blood cell poikilocytosis can occur, including problems in phospholipid metabolism (Dacie et al., 1975) or acanthocytosis in cases of liver cirrhosis (Cooper, 1980). Teardrop cells may also arise as a result of splenic macrophages removing precipitated hemoglobin or autophagocytic vacuoles from RBCs (Bike, 1993). Sickle cells have been identified in both hemoglobin sickle cell sickness and anoxia (Dacie et al., 1975). Crenation of red blood cells occurs as a result of cellular energy (ATP) depletion caused by dehydration or expansion of the outer membrane leaflets (Stello et al., 2003). The vacuoles detected in erythrocytes could be attributed to unequal hemoglobin distribution, and enlarged blood cells were identified as a symptom of necrosis. This conclusion was comparable to that of Soliman and Sayed (2020).

B-carotene in *Spirulina* improves the recovery of RBCs and minimizes cell lysis and changes (Sayed and Authman, 2018; Hamed et al., 2019b; Sayed et al., 2023a; Sayed et al., 2023b). *Spirulina platensis* includes iron and vitamins, which regulate red blood cell synthesis (Hemalatha, 2012), as well as polysaccharides, which promote RBC renewal (Mohamed et al., 2014). Furthermore, phycocyanin, a pigment, stimulates the erythropoietin (EPO) hormone, which is responsible for erythropoiesis (Zhang, 1994). However, recent data revealed that co-treatment with *Spirulina* did not significantly reduce the effects of PP-MPs, indicating that the *Spirulina* dose (200 mg/L) was insufficient.

PP-MPs have been described in the literature to be among the most prevalent types of plastics in aquatic environments, with a significant carbon peak in EDX analysis (Wang et al., 2017; Furfaro et al., 2022; Yedier et al., 2023). PP-MPs produce oxidative stress, organ damage, and decrease survival in *Danio rerio* and *Oreochromis mossambicus* in freshwater (Lei et al., 2018; Jeyavani et al., 2023). Furthermore, Jeyavani et al. (2023) showed oxidative stress, DNA damage, increased apoptosis, and histological alterations in *O. mossambicus* liver tissues subjected to a diet enriched with PP-MPs. Fish serum’s ALT and AST levels are used as markers of their hepatic functioning (Abdel-Latif et al., 2020), can serve as early warning signs of significant changes in stressed organisms, and are useful indicators of an animal’s overall health status (Garima and Himanshu, 2015). These enzymes were primarily located in the cytoplasm of the hepatocyte, but they were also released into the bloodstream in cases of liver damage (Amacher, 1998). Based on liver damage, in this study, some blood indicators (ALP, AST, and ALT) exhibited a non-significant (*p* ≥ 0.05) increase in the PP-MP-exposed group (0.14 mg/L) and a significant increase in the PP-MP (0.28 mg/L)-exposed group when compared to the control group. The results obtained were consistent with what was observed in catfish (*C*. *gariepinus*) after exposure to polystyrene nanoplastics (PS-NPs) (Sayed et al., 2021a; Abdelbaky et al., 2022; Soliman et al., 2023), after exposure to microplastic particles in African catfish (*C*. *gariepinus*), and after juvenile of common carp *C. carpio* were exposed to three distinct sizes of polyethylene plastics: nano, micro, and macro (Hamed et al., 2022).

Many fish species, such as the common goby (*Pomatoschistus microps*) (Oliveira et al., 2013), common carp (*C. carpio*) (Haghi Nematdoost and Banaee, 2017; Ammar et al., 2023), Nile tilapia (*O. niloticus*) (Hamed et al., 2019a), catfish (*C*. *gariepinus*) (Sayed et al., 2021a), and red tilapia (*O. niloticus*) (Yang et al., 2023), have all experienced changes in their biochemical parameters as a result of MP exposure. Serum biomarkers generally function as indicators of organ failure and biological process disruption. These biomarkers were still increased when *Spirulina* supplementation was used with (0.14 and 0.28 mg/L) PP-MPs, which indicated that a *Spirulina* dose of 200 mg/L was insufficient to treat the effects of PP-MPs. When these enzymes are released from the cell and increase in blood levels, they are thought to be markers of damage to the cell membranes (Banaee et al., 2014). Damage to hepatocytes, which causes detoxication and the hepatocytes’ subsequent export into the bloodstream, may be the result of these enzymatic alterations (Sayed et al., 2022b).

Increased blood concentrations of these enzymes signify pathological alterations in the permeability and/or cytotoxicity of hepatocyte cell membranes, implying the detrimental impact of PP-MPs on the integrity of the liver (do Nascimento et al., 2023). These results corroborated to those of Hamed et al. (2019a), who found a significant increase in juvenile *O. niloticus* exposed to microplastic and do Nascimento et al. (2023) who noted similar findings in Nile tilapia following ingestion of PP-MPs. According to Kim et al. (2021b), an elevation in AST may lead to hormonal imbalances, lipid metabolic disorders, and liver-related lipoproteins, ultimately resulting in elevated levels of cholesterol and triglycerides in fish. The disruption of cell membranes and mitochondria resulted in a significant increase in ALT levels in *C. carpio* exposed to MPs, as reported by Banaee et al. (2019). Another significant enzyme in fish metabolism is ALP, which is necessary for several physiological processes such as cell division, protein phosphorylation, and metabolite transport. Fish metabolism can therefore be impacted by variations in ALP activity (Chinnadurai et al., 2022). Additionally, Haghi Nematdoost and Banaee (2017) found a large increase in ALP levels when *C. carpio* was exposed to MPs, and they hypothesized that this was due to hepatocyte necrosis and degeneration caused by MP toxicity.

Karami et al. (2016) highlighted the significant role of the liver as the organ responsible for metabolizing fats and describe how microplastics can impair its functioning. In the present work, the liver tissue from *C. gariepinus* exposed to PP-MPs (0.28 mg/L) showed different levels of damage, such as dilated central vein full of red blood corpuscles and leucocytes, deformation and severe degeneration in hepatic structures, necrosis of the main bulk of hepatic cells, vacuolated cells with pyknotic nuclei with fine heterogamous acidophilic in the cytoplasm, and increased necrotic areas, while the liver tissue from the PP-MP (0.14 mg/L)-exposed group exhibited little pathological changes compared to the the PP-MP (0.28 mg/L)-exposed group. These results indicated that these pathological changes increased with PP-MP concentration, suggesting that the effect of PP-MPs on liver tissue was dose-dependent. In support of our results, Jeyavani et al. (2023) reported that PP-MP exposure showed that the degeneration of liver cells was directly correlated with the exposure doses to PP-MPs, as evidenced by a decrease in sinusoid infiltration with leukocytes, hepatocyte necrosis, hepatocyte vacuole formation, and dilated sinusoidal atrophy. In addition, according to Espinosa et al. (2019), European sea bass can sustain liver tissue damage after up to 3 weeks of exposure to polyvinylchloride and polyethylene microplastics. In accordance with, Jeyavani et al. (2023), the present work showed that the catfish (*C. gariepinus*) that were exposed to PP-MPs for up to 15 days produce ROS levels that cause oxidative stress, which damages the liver tissues. Co-treatment with *Spirulina* decreased these detrimental effects on liver function, possibly as a result of its capacity to scavenge and suppress ROS (Kuriakose and Kurup, 2011; Soliman et al., 2021).

After 45 days of recovery under normal conditions, it was clear that there was a considerable improvement in the percentage of poikilocytosis and nuclear abnormalities of RBCs, as well as a non-significant improvement in hemato-biochemical parameters and liver tissue. This indicated the threat of PP-MPs, which typically end up in the environment and persist in freshwater ecosystems. The current study’s findings showed that the morphological restoration of *C. gariepinus* liver tissue took longer than the recovery of hemato-biochemical parameters. These seem to be significant adaptive reactions of PP-MP-intoxicated fish, implying the potential compensatory responses of other organic systems that enable fish to rapidly return to normal plasma ion concentration and blood parameters, as reported by Cerqueira and Fernandes (2002) after recovery from copper exposure of the tropical fish *Prochilodus scrofa* and Amorim et al. (2023) after recovery in *O. niloticus* fish exposed to urban effluents. Nonetheless, metabolic costs to maintain homeostasis can exceed the critical level for fish and have serious consequences for the energy balance, reproduction, and survival of the fish (Cerqueira and Fernandes, 2002). A defense state that preserves the hepatocellular membrane’s structural integrity is presented by a reduction in ALT and AST activity during recovery (Pari and Amali, 2005). This fact was linked to the improvement in liver cell damage and necrosis during recovery treatments due to the absence of toxic stress (Meraj et al., 2017).

## 5 Conclusion

Exposure to PP-MPs at 0.14 and 0.28 mg/L concentrations for 15 days showed deleterious effects, resulting in disruption in hematological and biochemical parameters, alteration in erythrocyte profile, poikilocytosis, and nuclear abnormalities of RBCs and changes in the liver functions. The current study outcomes displayed that, despite a 45-day recovery period, the effects of PP-MPs were not completely lost after the recovery period, indicating the threat of PP-MPs released from various sources, particularly paper cups, which are widely used in our daily lives and typically end up in the environment and persist in freshwater ecosystems. This reflects the adverse effects of PP-MPs on aquatic biota, particularly fish, and may get wider attention from scientists due to the potential health implications. The *Spirulina* (200 mg/L) dose was insufficient to significantly treat the effects of PP-MPs, and its effects disappeared after exposure. Thus, *Spirulina* microalga must be widespread at a high concentration in aquatic environments for contribution of removal or protection against the toxicity of PP-MPs, which have deleterious effects on aquatic organisms.

## Data Availability

The original contributions presented in the study are included in the article/Supplementary material; further inquiries can be directed to the corresponding author.
